# Injury of African American Women by Financial Exploitation

**DOI:** 10.1093/geroni/igaa057.2459

**Published:** 2020-12-16

**Authors:** Kimethria Jackson, Patsy Smith, Janet Wilson

**Affiliations:** 1 University of Oklahoma Health Sciences Center, Oklahoma City, Oklahoma, United States; 2 University of Oklahoma Health Sciences Center, Reynolds Center for Geriatric Nursing Excellence, Oklahoma City, Oklahoma, United States

## Abstract

Financial exploitation (FE) is one of the most common forms of older adult mistreatments. The World Bank defines FE as financial violence and the World Health Organization describes FE as financial or material abuse. Both international organizations recognize that FE causes deprivation/neglect leading to physical and emotional injury to victims. Older African Americans (OAA) are disproportionately affected by FE, impacting their health and welfare. A qualitative phenomenological study explored the lived experiences of FE among OAA. A Community Based Participatory Research approach was used to partner with a predominately African American-faith based community. Participant recruitment (n=12) was through community sponsored seminars that included verbal presentations, group discussions; individual surveys, Older Adult Financial Exploitation Measure (OAFEM). OAFEM data was analyzed to identify risks. Analysis of the interviews included Open and Axial coding, and data categorization. Using NVivo, Stevick-Colaizzi-Keen analysis as modified by Moustakas, five themes emerged.

